# Insights on prospects of nano-siRNA based approaches in treatment of Cancer

**DOI:** 10.3389/fphar.2022.985670

**Published:** 2022-08-25

**Authors:** Rajat Goyal, Hitesh Chopra, Inderbir singh, Kamal Dua, Rupesh K. Gautam

**Affiliations:** ^1^ MM School of Pharmacy, MM University, Sadopur-Ambala, Haryana, India; ^2^ MM College of Pharmacy, MM (Deemed to be University), Mullana-Ambala, Haryana, India; ^3^ Chitkara College of Pharmacy, Chitkara University, Patiala, Punjab, India; ^4^ Discipline of Pharmacy Graduate School of Health Faculty of Health, Australian Research Centre in Complementary and Integrative Medicine (ARCCIM) University of Technology Sydney, Sydney, NSW, Australia

**Keywords:** cancer, siRNA, gene silencing, exosomes, nanoparticles, nanomaterials

## Abstract

siRNA interference, commonly referred to as gene silence, is a biological mechanism that inhibits gene expression in disorders such as cancer. It may enhance the precision, efficacy, and stability of medicines, especially genetic therapies to some extent. However, obstacles such as the delivery of oligonucleotide drugs to inaccessible areas of the body and the prevalence of severe side effects must be overcome. To maximize their potential, it is thus essential to optimize their distribution to target locations and limit their toxicity to healthy cells. The action of siRNA may be harnessed to delete a similar segment of mRNA that encodes a protein that causes sickness. The absence of an efficient delivery mechanism that shields siRNA from nuclease degradation, delivers it to cancer cells and releases it into the cytoplasm of specific cancer cells without causing side effects is currently the greatest obstacle to the practical implementation of siRNA therapy. This article focuses on combinations of siRNA with chemotherapeutic drug delivery systems for the treatment of cancer and gives an overview of several nanocarrier formulations in both research and clinical applications.

## Introduction: Cancer and siRNA-based therapy

Cancer is a dangerous illness that threatens both human health and existence because of its complexity, which includes several factors, multiple genes, and multiple pathways. It is the second greatest cause of mortality in the globe, placing a heavy load on the healthcare system. Cancer is a deadly, life-threatening illness that affects millions of people throughout the globe, and early identification is the key to curing it (Sharma et al., 2020; Singla et al., 2021). Cancer society stated that nearly 14.1 million cases of cancer and approximately 8.2 million deaths have been reported, implying that one out of every seven deaths is caused by cancer, which is greater than consolidated tuberculosis, Acquired Immune Deficiency Syndrome (AIDS), and protozoal infections. With this trend, it is expected that by 2030, around 21.6 million new cases of cancer and approximately 13.0 million deaths due to cancer would arrive (Sharma et al., 2021).

Cancer can spread throughout organs in the body that differ from its primary site of origin, and is primarily caused through DNA (deoxyribose nucleic acid) mutations that occur intracellularly (Bhattacharya et al., 2022). Cancer often disrupts cell-to-cell communication, resulting in crucial gene malfunction. During the cell cycle, this aggravation continues, resulting in aberrant cell growth. Prostate, lung, colon, bronchi, urinary bladder, and rectum cancers affect the majority of males. The breast, lungs, colon, bronchi, rectum, thyroid, and urinary corpus are the most frequent cancer sites in women (Hassanpour and Dehghani, 2017; Kanugo et al., 2022). Cancer is a significant target for RNA interferences (RNAi)-based therapeutic approaches, but the major contest in cancer rehabilitation is identifying and targeting the metastatic cells, that has been spreading from the original tumor. The jury is still out on whether small interfering RNA (siRNA) can be effectively delivered to such cells and whether appropriate targets can be identified to destroy the metastatic population (Kim and Rossi, 2007). Furthermore, in some cases, activation of immune system in conjunction with siRNA-mediated silencing of genes may be anticipated, resulting in improved therapeutic efficacy. As a result, siRNAs comprising potent immunostimulatory motifs may be employed for dealing with tumors and viral infections (Marques and Williams, 2005).

RNA interference (RNAi) is a significant biological mechanism in which the existence of double-stranded RNA (dsRNA) inhibits the specific gene expressions with a homologous sequence to the dsRNA (Oh and Park, 2009). There are two types of RNA interference: small integrated RNAs, or siRNAs, and micro RNAs, or miRNAs, which are formed from poorly paired non-coding hairpin RNA structures. In medical and pharmaceutical research, the siRNA is a double-stranded RNA-based fragment that has a considerable deal of therapeutic promise. Some clinical studies are now testing numerous possible siRNA candidates to treat respiratory illnesses, and cancer at this moment. While siRNA silences genes by cleaving precisely complementary mRNA, microRNAs arbitrate transcript deprivation and translational suppression for targets that aren’t completely complementary (de Fougerolles et al., 2007). As compared to conventional clinical therapies, gene therapy exhibits higher sensitivity and specificity in terms of prompting tailored gene expressions, which display the extraordinary changes in the progression of metastasis, tumorigenesis, and immune responses (Gao et al., 2021).

Nontarget gene silencing may result in data interpretation issues as well as potential noxiousness. To evade this problem, designing and selecting the potent siRNAs ought to be done with due care. The basic parameters for the selection of siRNAs include the deliberation of internal-recurring sequences, guanine-cytosine (GC) content, secondary structures, appropriate siRNA length (i.e., 19–22 bps), and base preference at the explicit positions in the sense strand. The immune stimulation is another challenge faced by siRNA gene therapy. The incorporation of too much siRNA leads to non-specific proceedings owing to the initiation of innate immune responses. Interferons and inflammatory cytokines have been persuaded *via* Nuclear factor kappa (NF-_k_B) activation, and interferon-regulatory factors, after the siRNA recognition by toll-like receptors such as TLR7, TLR8, and TLR9 (Oh and Park, 2009).

### Mechanism of gene silencing by siRNA

**FIGURE 1:**
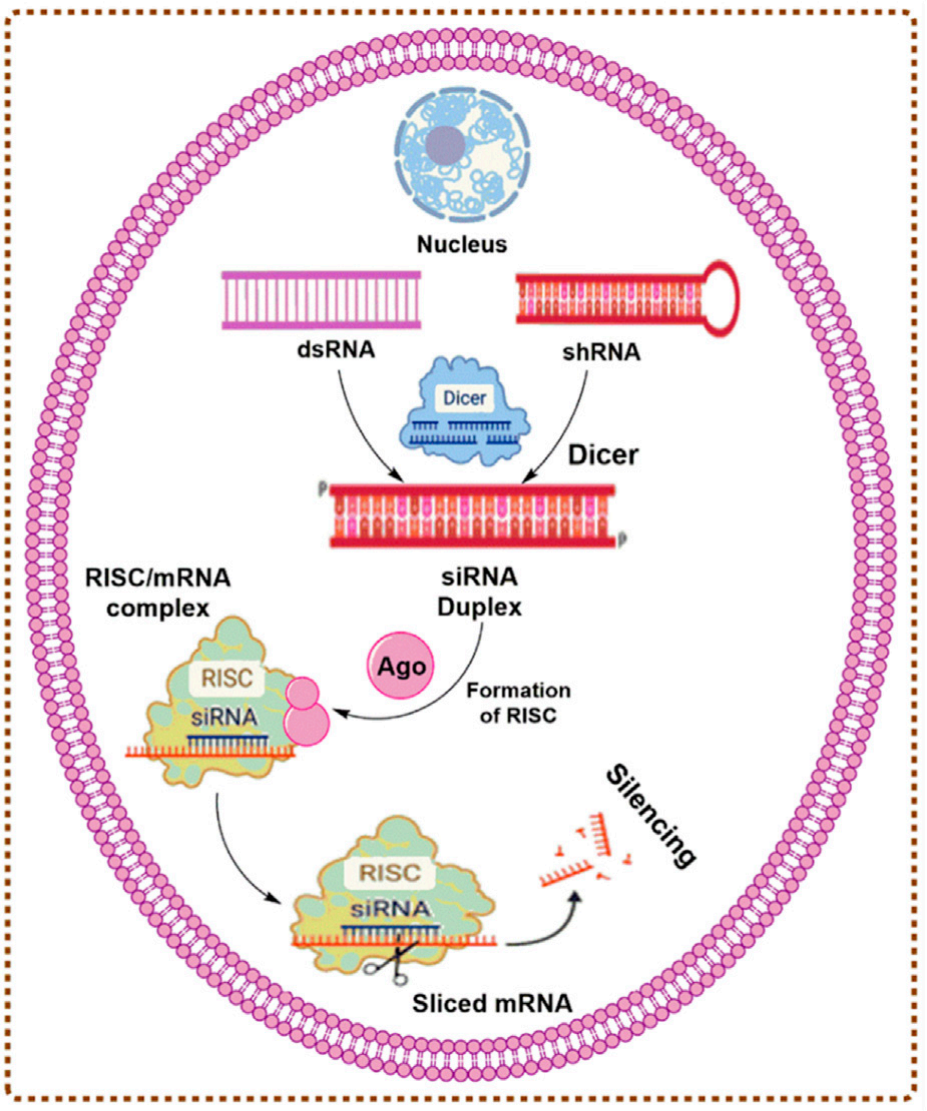
The mechanism of gene silencing by siRNAs.

The gene silencing demonstrates the potential of effectiveness in both tumor cells and stromal cells via inhibition of tumor-promoting genes (Vetvicka et al., 2021). Mutated cancer suppressor genes, oncogenes, and several other genes involved in the formation of cancers are advantageous gene silencing targets using RNAi-based techniques due to their distinct functional mechanism, increased potential, and specificity of RNAi-based gene silencing. The production of siRNA occurs in two steps. During the initiation step, Dicer cleaves a lengthy double-stranded RNA (200–500 bp) into pieces of 21–23 nucleotides, producing siRNA. In the affecting phase, helicase divides the double-stranded siRNA, following which the endogenous endonucleases define the sense-strand and the antisense strand guides the RNA-induced silencing complex (RISC) to the target mRNA. As a member of the RISC, Argonate (Ago) destroys this mRNA through ribonuclease activity in the piwi region. As long as RISC continues to degrade the target mRNA, gene expression is halted. mRNA may be degraded in two distinct ways. First, ribonuclease might degrade them, and then RNA polymerase would create double-stranded RNA and bind it to the homologous strands, resulting in the continuous interference process (Mansoori et al., 2014; Sharma et al., 2022). The mechanism of gene silencing by small interfering RNAs (siRNAs) (Majumdar et al., 2017) is described in Figure 1.

## Role of siRNA-based therapy in the treatment of Cancer

The accessibility of the human genetic sequences has revolutionized the strategies of employing nucleic acids with complimentary sequences to explicit the target genes, and to promote the drug discovery methodologies and target validation (Devi, 2006). Dysregulation of gene expression is a common characteristic of cancer. The potential therapeutic use of suppressing an oncogene’s production at the mRNA level, as opposed to the gene product’s activity, has sparked the researchers’ curiosity for quite some time. Therefore, RNAi provides interesting new potential to specifically target genes that are dysregulated in a variety of diseases, including cancer. RNA interference (RNAi) is a gene-silencing technology with great potential in cancer therapy (Ali et al., 2012).

siRNA feats endogenous RNAi circuits, enabling the explicit reduction of disease-linked genes, and may be administered to any gene with a complementary sequence (Leung and Whittaker, 2005). Numerous targets are anticipated for siRNA-arbitrated gene silencing, which may improve the potential of the immune system toward cancer (Ghafouri-Fard and Ghafouri-Fard, 2012). The main goal of siRNA-based therapy is to treat cancer via silencing the genes, that are important for tumor development or drug resistance. As a result, one of the rudiments for RNAi-based therapy is the selection of appropriate gene targets. A candidate gene should ideally: 1) important for the progression of tumor or drug resistance, 2) preferentially expressed in targeted tumors, and 3) associated with reliable biomarkers, utilized to measure the clinical and biological responses to RNAi therapy (Wang et al., 2017). Several siRNAs are undoubtedly designed to target cancerous oncogenes, including viral oncogenes, dominant oncogenes, and improperly regulated oncogenes. In addition, siRNAs have been intensively studied for their propensity to quiet target genes that are essential for tumor-host interactions and radiation or chemotherapy resistance. Significant apoptotic or anti-proliferative effects were seen when siRNAs were utilized to silence cancer-associated risk genes (Pai et al., 2006).

Cancer growth, metastasis, and invasion are primarily controlled by angiogenesis. The vascular endothelial growth factor (VEGF) family has been identified as a crucial mediator of these endothelial cell changes seen in several cancer types. By inhibiting VEGFR-1 and VEGFR-2 using siRNAs, angiogenesis might be prevented. The Wingless-related integration site (Wnt) pathway plays a crucial part in the carcinogenic process. The suppression of Wnt pathways by siRNAs may serve as a potential anticancer drug since it has been utilized to target malignant stem cells that respond poorly to chemotherapy (Chen and Huang, 2008). Novel delivery strategies are needed to improve siRNA stability, tumor cell selectivity, non-specific immunostimulatory effects, and off-target effects. These drug delivery systems must be adapted for various tumors since the administration route may vary (Dana et al., 2017). The description of the siRNA-based delivery systems in cancer treatment is summarized in Table 1.

**TABLE 1:** Examples of siRNA delivery systems in the treatment of cancers.

S. No	Delivery Systems	Property	Target gene	Animal model	Type of study	References
1	Liposome	Cationic cardiolipin liposome	Raf-1	Prostate cancer xenograft	Down-regulation of gene expression *in vivo*	Pal et al. (2005)
2	Liposome	Liposome-polycation-DNA	EGFR (Epidermal Growth Factor Receptor)	Lung cancer xenograft	EGFR silencing induces apoptosis, cell cycle arrest, tumor cell growth inhibition, and chemosensitization *in vitro* and *in vivo*	Li et al. (2008)
3	Liposome	Neutral liposomes (DOPC)	EphA2 (Ephrin Receptor A2)** **	Ovarian cancer xenograft	Primarily induce carcinogenic potential through higher levels of the unphosphorylated form *in vivo*	Landen et al. (2005)
4	Liposome	Immuno-liposome	HER-2 (Human Epidermal Growth Factor Receptor 2)	Breast cancer xenograft	Inhibition of HER-2 expression *in vivo*, and induce apoptosis in human breast carcinoma tumors	Hogrefe et al. (2006)
5	Liposome	Cationic liposome	CD31 (Cluster of differentiation 31)	Prostate cancer xenograft	Silencing of tumor-causing genes *in vivo*	Santel et al. (2006)
6	Liposome	SNALP (Stable Nucleic Acid Lipid Particles)	HBV (Hepatitis B virus)	HBV vector-based mouse model	Potent and persistent *in vivo* anti-HBV activity of chemically modified siRNAs	Morrissey et al. (2005)
7	Liposome	Neutral liposomes (DOPC)	IL-8 (Interleukin 8)	Ovarian cancer xenograft	IL-8 gene silencing decreases tumor growth *in vivo* through anti-angiogenic mechanisms	Merritt et al. (2008)
8	Polymer	Polyester amine (PEA)	Akt1	Urethane-induced lung cancer	Suppression of lung tumorigenesis *in vivo*	Xu et al. (2008)
9	Polymer	Polyethylenimine (PEI)	PTN (Pleiotrophin)	Orthotopic glioblastoma	Exerts antitumoral effects in glioblastoma xenografts *in vivo*	Grzelinski et al. (2006)
10	Polymer	Polyethylenimine (PEI)	HER-2	Ovarian cancer xenograft	Reduction of tumor growth *in vivo* via siRNA-mediated HER-2 downregulation	Urban-Klein et al. (2005)

## Lipid-based approaches for siRNA delivery

For siRNA delivery, lipid-based nanoparticles are among the most researched. Experiments conducted on them revealed that these nanoparticles were the most promising for systemic siRNA delivery. Thus, it is simple to build nanoparticles such that the siRNA they carry accumulates at specific sites and is not rapidly removed or degraded by the body. There are several types of lipid-based nanoparticles, including liposomes, niosomes, solid lipid nanoparticles, and micelles. Hydrophilic siRNA molecules in the aqueous core of liposome nanoparticles are insulated from nuclease activity, increasing their stability and half-life. Encapsulating siRNA molecules helps them enter cells (Buyens et al., 2012; Zhang et al., 2014).

The study of liposomes has led to the invention of several manufacturing techniques, including the thin-film hydration method, the heating method, and the ether injection technique. To optimize the delivery of their therapeutic payloads, these nanoparticles may now be manufactured using a range of compositions and processes, allowing for more control over the essential liposome features such as particle size and surface charge (Allen and Cullis, 2013). The liposomes’ surface charge will influence their dispersion and interaction with biological surfaces. Cationic, anionic, or neutral surface charges may all be achieved using liposomes. Phospholipids employed in the creation of liposomes will determine the final net surface charge.

Cationic liposomes may be made using cationic phospholipids such as dioleoyl phosphatidylethanolamine, 1,2-dioleoyl-3-trimethylammonium-propane (DOTAP), dioleoyl phosphatidylethanolamine (DOPE), oleic acid (OA), and dimethyl-dioctadecyl ammonium bromide (DDAB). For these liposomes to work, the medicine must have a negative charge, which will result in the development of stable complexes. Lipoplexes are the cationic liposomes and the negatively charged medicines that form these complexes (Lee et al., 2013; Ozpolat et al., 2014). As the siRNA molecules have a strong negative charge and will thus combine with the cationic liposomes, the distribution of the siRNA to the target location will be boosted and remain unaltered. Liposome nanoparticles that have a cationic charge on their surface are more likely to be taken up by the target cells because of their increased contact with the negatively charged cell membrane (Laouini et al., 2012).

However, specific toxicity issues must be addressed before employing these cationic liposomes for medication delivery purposes. Cationic liposomes have been shown to cause hepatotoxicity and lung inflammation at varying doses, according to many studies (Urban-Klein et al., 2005; Ozpolat et al., 2014). After interacting with serum proteins such as opsonins, the reticuloendothelial system identifies cationic liposomes, resulting in rapid macrophage clearance of these nanoparticles. Using cationic liposomes in lipoplexes with negatively charged siRNA, Lipopolymers can encapsulate and transfect siRNA with excellent efficiency. According to the literature, this is the case. In the study of Chae et al., antisphingosine-1-phosphate receptor-1-siRNA was synthesized and complexed with DOTAP-based cationic liposomes. When transfected into lung cancer cells, these lipoplexes dramatically lowered the expression of the target protein (Chae et al., 2004). A lipoplex containing anti-GADPH (glyceraldehyde-3-phosphate dehydrogenase) siRNA was also made using cationic liposomes aimed at P-selectin, a molecule involved in adhesion in cells. Lipoplexes has ability to protect siRNA molecules and to deliver them effectively to target cells and also can supress GADPH gene (Constantinescu et al., 2019). Hattori et al. (2019) employed cationic lipids to create siRNA-encapsulated liposomes. In a mouse model of lung cancer, cationic liposomes carrying siRNA targeting protein kinase N3 (PKN3) reduced tumor growth . Stabilized nucleic acid-lipid particles (SNALPs) are cationic liposomes produced by Tekmira Pharmaceuticals Corporation for the systemic administration of siRNA. These liposomes use a variety of ionizable cationic lipid components. Nanoparticles loaded with siRNA are highly resistant to external conditions and efficiently transport siRNA to target cells (Gomes-Da-Silva et al., 2012; Shrivastava et al., 2020).

An anionic surface charge may be achieved by using anionic phospholipids. Naturally occurring anionic phospholipids include phosphatidylglycerol, phosphatidylinositol, and phosphatidic acid (Draz et al., 2014). Anionic liposomes, which are less toxic than cationic liposomes, have also been extensively studied as a potential siRNA delivery vector (Tam et al., 2013). Using phosphatidylethanolamine-based anionic liposomes, Yu and others were able to deliver VEGF-targeting siRNA. Flow cytometry and Western blotting findings indicate that the loaded anionic liposomes may transport siRNA into cells and inhibit VEGF expression (Yu et al., 2019). Due to charge repulsion, reduced encapsulation effectiveness of siRNA into anionic liposomes may ensue if the siRNA molecules contain a negative charge. Calcium ions and other divalent cationic bridge ions were shown to be effective in solving this problem (Urban-Klein et al., 2005; Hogrefe et al., 2006). Using calcium ion bridges, Kapoor et al. were able to encapsulate siRNA with an anionic liposome with 99 percent efficiency (Kapoor and Burgess, 2012).

As an alternative to employing charged liposomes because of safety concerns about their toxicity (Urban-Klein et al., 2005; Zhang et al., 2014) thus to make neutral liposomes, researchers have recommended employing neutral phospholipids such as dioleoyl-n-glycerol-phosphatidylcholine (DOPC). As a nanoparticle drug delivery method, neutral liposomes have an excellent safety profile and minimal interaction with anti-inflammatory opsonin, resulting in extended nanoparticle circulation durations. Due to these advantages, several researchers have used neutral liposomes for the administration of various pharmaceuticals. Several medications, like Doxil, an FDA-approved pharmaceutical comprising neutral liposomes carrying doxorubicin, have been put on the market as a consequence (Obeid et al., 2018). DaunoXome is yet another FDA-approved medication for the administration of daunorubicin through neutral liposomes. The EphA2 oncoprotein and the cytokine interleukin 8 (IL-8) were both targeted by Merritt et al. utilizing neutral liposome formulations to deliver siRNA. On the ovarian cancer-bearing animal, they evaluated this technique and found an efficient gene knockdown (Morrissey et al., 2005).

Lipofectamine and Oligofectamine are often used for siRNA transfection. Liposomes are the second-most-used siRNA delivery method after lipofectamine and Oligofectamine. Liposomes, impermeable phospholipid bilayer-encased aqueous core containers, may hold both lipophilic and hydrophilic medicines. Hydrophilic lipid bilayers encase lipophilic molecules like genes and siRNA. By changing its composition, size, and charge, liposomes may circulate and encase malignant cells. Liposome PEGylation promotes circulation retention (Fang et al., 2006; Romberg et al., 2008). Liposomes’ lipids may be coupled with certain ligands to improve their target selectivity (Guo et al., 2010). The capacity of cancer stem cells to metastasize, propagate and resist chemotherapeutic treatments has made CD44 expression on tumor cells a biomarker of cancer stem cells (Jaggupilli and Elkord, 2012). An association between CD44 expression and the JAK2/STAT3 signaling pathway may play an important role in TNBC’s aggressiveness (Ma et al., 2014). Liposomal nanocarriers have been developed by Alshaer et al. (2018) for the delivery of siRNA against MDA-MB-231. Luc-2 siRNA was successfully loaded into a liposome containing an anti-CD44 aptamer. Researchers first encased siRNA in PEGylated liposomes containing siRNA-condensed protamine peptides to disseminate siRNA. Any residual siRNA on the liposome surface was removed by enzymatic digestion. After siRNA was encapsulated in PEGylated liposomes, Aptamer1 was added. 60 to 80 percent siRNA entrapment efficiency has been shown for PEGylated liposomes at 2 nmol siRNA concentration. It was shown that aptamer-1 siRNA/protamine-liposome complexes resulted in a higher degree of gene silence in MDA-MB-231 cell lines than siRNA/liposome complexes without aptamer-1, perhaps due to the liposomes’ enhanced absorption (Alshaer et al., 2018). Deng et al. proposed new liposome-based nanocarriers for the simultaneous delivery of siRNA and Doxorubicin in another study (Dox). The siRNA film was positioned atop the dox, which was then decorated with a targeting moiety. The medication and siRNA were successfully delivered to their designated areas using this delivery system. An *in vitro* examination of the nanocarrier’s capacity to carry dox revealed its efficacy, and tumor volume dropped to one-eighth of that of the control group. In addition, it does not indicate any toxicity, enabling patients to have more therapy options available. This layer-by-layer technique offers tremendous promise for the delivery of siRNA and Dox in the battle against aggressive and resistant triple-negative breast cancer (Deng et al., 2013).

Fork head transcription factor (FOXM1) was shown to be crucial in the development of chemoresistance and the decrease in susceptibility to anticancer medicines (Abnous et al., 2018). FOXM1 overexpression has been linked to a worse survival rate in patients (Bektas et al., 2008). FOXM1 also plays a role in Dox resistance via genetic alteration (Park et al., 2012). Single-stranded DNA-aptamers have been created that can effectively decrease FOXM1 transcriptional activity (Xiang et al., 2017). In cancer cells, FOXM1apt mainly binds to FOXM1 and inhibits FOXM1’s transcriptional activities (Abnous et al., 2018). Ghandhariyoun et al. have created a liposomal structure based on FOXM1 aptamers to deliver dox to breast cancer cells that are resistant to treatment. While monotherapy was less effective, the combination of liposomes, dox, and FOXM1 aptamer boosted cytotoxic activity, perhaps as a result of the enhanced uptake of dox by liposomes (Ghandhariyoun et al., 2020). To put it another way, liposomes have a lot of promise as a nanocarrier to carry anticancer medicines to treat triple-negative breast cancer.

### Exosomes

Most cell types in the body release exosomes, a class of nanoparticles with a diameter of 30–120 nm (Record et al., 2011; Vlassov et al., 2012). Blood, urine, amniotic fluid, saliva, and cerebrospinal fluid are just a few of the extracellular fluids from which they may be extracted (Vlassov et al., 2012; Witwer et al., 2013; Jia et al., 2014). To begin the production of exosomes, endosomal membranes are first invaginated, creating multivesicular bodies (MVBs) (Raposo et al., 1996). Exosomes did not have characteristic to shed micro vesicles to originate itself to cell membrane. Since lysosomal surface proteins including LAMP (Lysosome-associated membrane glycoproteins) and CD63 are present in the exosomal membrane, the development of MVBs during exosome genesis reveals some parallels with the MVBs generated during lysosome genesis (Riteau et al., 2003; Caby et al., 2005). The so-called endosomal sorting complex needed for transport (ESCRT), which is also critical for lysosome formation (Raiborg and Stenmark, 2009; Baietti et al., 2012), is essential for the production of exosomes and the sorting of cargo into them. A total of four main protein complexes, designated as ESCRT-0–ESCRT-III, comprise the ESCRT machinery. To facilitate the release of ubiquitinylated proteins in nanoparticles like exosomes, the ESCRT machinery and many auxiliary proteins are known to enhance endosomal sorting.

In several studies conducted in recent years, exosome-based medication delivery systems have been proven to alleviate disease states, including different cancer models. The development of an exosome-based drug delivery system requires the selection and control of several components to achieve maximum performance and efficacy. Due to the fast degradation of interfering RNA molecules in the circulatory system, exosome-based transport of these RNA molecules is of significant interest (Tabernero et al., 2013). Efficient delivery of MAPK1-siRNA into recipient peripheral blood mononuclear cells was achieved using exosomes from both cells and plasma (Wahlgren et al., 2012). Both RAD51 and RAD52 siRNA were delivered using the same manner to induce gene knockdown and reduce fibrosarcoma cell survival and proliferation (Shtam et al., 2013). GAPDH and BACE1 mRNA expression was downregulated in neurons following targeted administration of siRNA-enriched exosomes (Alvarez-Erviti et al., 2011). The use of short hairpin RNAs (shRNAs) and so-called self-delivering RNAs as therapeutic cargo in exosomes has been discussed in several publications (Pan et al., 2012; Shtam et al., 2013). It has been shown that shRNA-loaded exosomes may reduce HCV infection in liver cells by targeting viral entry receptors and the hepatitis C viral replication machinery (Pan et al., 2012).

Metastasis-related proteins that promote tumor growth and metastasis include S100A4, which is a member of the S100 family (Boye and Mælandsmo, 2010; Mishra et al., 2012; Grum-Schwensen et al., 2015; Dahlmann et al., 2016; Liu et al., 2019a). Upregulated S100A4 might provide a favorable milieu for malignant breast cancer cells in addition to the establishment of the pre-metastatic niche (PMN) (Boye and Mælandsmo, 2010; Mishra et al., 2012; Grum-Schwensen et al., 2015). S100A4 has eluded effective treatment for far too long. Simultaneous induction of post-transcriptional gene silencing by siRNA, and RNA interference (RNAi) has the potential to be used in the treatment of genetic diseases and cancer. Silencing the expression of S100A4 by siRNA (siS100A4) is well documented (Wilson and Doudna, 2013; Grum-Schwensen et al., 2015).

**FIGURE 2:**
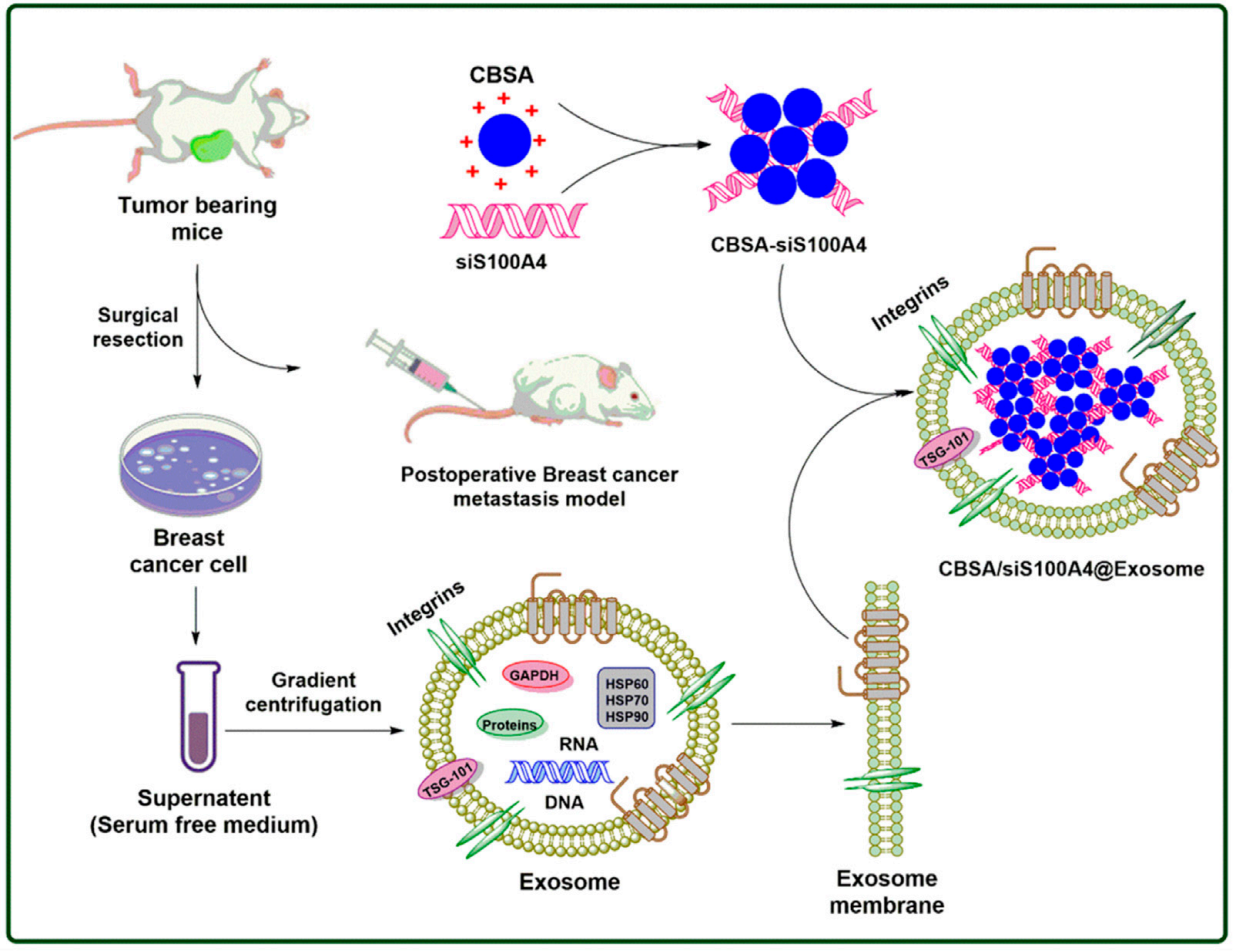
Postoperative breast cancer metastatic suppression via exosome-mediated siRNA delivery.

Biomimetic nanoparticles (CBSA/siS100A4@Exosome) with a CBSA/siS100A4 core and an exosome membrane shell have been studied by researchers using cationic bovine serum albumin (CBSA) (as shown in Figure 2) (Zhao et al., 2020). Postoperative inhibition of lung metastases by the CBSA/siS100A4@Exosome biomimetic was remarkable, and the biomimetic effectively accumulated in the lungs and permitted gene silencing. Due to its non-toxic, non-antigenic, and biodegradable characteristics, serum album has been widely exploited as a drug delivery medium (Liu et al., 2019b). Using cationic amino groups to alter bovine serum albumin, new activities were added to the protein without affecting its structure. On account of CBSA’s positive charge, which makes it easier for it to pass exosome membranes, the optimum pI values of CBSA have been widely studied as a siRNA carrier (Bickel et al., 2001; Han et al., 2014). Furthermore, CBSA/siRNA@Exosome showed excellent biocompatibility and very robust suppression of lung metastases as a result of the elevated accumulation in pulmonary PMN of siRNA via organotropopism mediated by exosome membranes.

Until recently, HeLa, HEK-293, and murine melanoma cell lines were used to make exosomes. (Lee et al., 2011; Ohno et al., 2013; Takahashi et al., 2013). Due to their surface-protein composition, immature dendritic cells are good exosome donor cells (Yin et al., 2013). The labor required to scale up their manufacture and low exosome yield limit their clinical translatability (Tian et al., 2014). Mesenchymal stem cells (MSCs) offer superior exosome donor cells (Katakowski et al., 2013; Munoz et al., 2013) since they generate bigger numbers of exosomes and hence are ideal for therapeutic translatability (Chen et al., 2011). MSC exosomes and microvesicles may stimulate cancer, hence they should not be used in cancer therapy (Yeo et al., 2013).

Researchers found bovine milk as a suitable source for exosomes that might function as a medication delivery platform due to the limitations of cell-derived exosomes (Aqil et al., 2019). Bovine milk exosomes are a cost-effective, biocompatible, tumor-targeting, and non-toxic alternative, according to a new study. Due to their acidic stability, milk exosomes may be a better oral drug delivery vehicle (Melnik et al., 2014; Aqil et al., 2017a). Researchers have proven that milk-derived exosomes absorb, distribute, and transport small chemicals. Bovine milk exosomes, which are biocompatible and scalable nanoparticles (Munagala et al., 2016; Aqil et al., 2017a; Aqil et al., 2017b), are capable of carrying exogenous nucleic acids and, in cell culture assays, inducing target gene silencing and antitumor activity against lung carcinoma xenograft. Oral administration of milk-derived exosomes is more effective in inhibiting tumor growth in xenografts derived from lung and ovarian malignancies, indicating that the drug concentration within the tumor is higher. Exosomes alone had a negligible effect on wild-type mice in a previous study (Munagala et al., 2016), while bovine milk exosomes were shown to have no systemic or immunotoxic effects on wild-type mice (Agrawal et al., 2017).

Research by others (Sun et al., 2010; Kamerkar et al., 2017) has demonstrated that exosomes can withstand the severe environment of the stomach and are stable at low pH levels. The distribution of macromolecules through exosomes has been used just a few times before. Exosomes from immature murine dendritic cells were electroporated by Alvarez-Erviti et al. (Alvarez-Erviti et al., 2011) to transport siRNA. *In vitro* and *in vivo* delivery of siRNA to neurons, microglia, and oligodendrocytes in the mouse brain have been shown. They also show that siRNA administration to wild-type mice results in a considerable drop in the brain-amyloid levels. Exosomes produced from normal fibroblast-like mesenchymal cells have recently been extensively examined by Kamerkar et al. (2017). Exosome-loaded exosomes decreased pancreatic cancer in various mice models and dramatically improved overall survival, according to the researchers. Moreover, this research found that exosomes with the CD47 signal remained in the circulation of mice longer than liposomes, which is likely related to the protection of exosomes from monocytes and macrophages.

Alvarez-Erviti et al. (2011) have shown the efficient use of exosomes in siRNA delivery. Mice dendritic cells have been transfected with the exosomal membrane protein Lamp2b and the neuronal-targeting ligand RVG (Rabies Virus Glycoprotein). The cells generated exosomes that contained this protein. We recovered exosomes using electroporation, processed them, and then loaded them with siRNA directed against BACE1, a critical protein in the genesis of Alzheimer’s. Three days after intravenous injection of modified exosomes, BACE1 mRNA in the cerebral cortex of wild-type mice was found to be 60 percent lower. Injection of the modified exosomes did not increase blood levels of interleukin-6, interferon gamma-induced protein, tumor necrosis factor-alpha, or interferon-alpha, showing that the modified exosomes were immunologically inert. Alvarez-Erviti et al. used a biotechnological technique to construct exosome-based drug delivery devices that exhibited efficient *in vivo* siRNA dispersion. In 2012, Wahlgren and others successfully injected human exosomes with exogenous siRNAs and used them to deliver siRNA to human mononuclear blood cells *in vitro* (Wahlgren et al., 2012). To silence the MAPK-1 gene specifically, plasma exosomes transported the siRNA to the target cells with high efficiency. Exosomes have been used in several ways to transport siRNA *in vitro* and *in vivo* (El-Andaloussi et al., 2012; Kooijmans et al., 2012; Koppers-Lalic et al., 2013).

It was then determined that exosome-delivered siRNA was functional and produced post-transcriptional gene silence in recipient cells. Exosomes carrying siRNA against the RAD51 transcript down-regulated the RAD51 gene in HeLa and HT1080 cells co-cultured with exosomes. Researchers found that exosomes transported siRNA to target cells, which silenced certain genes and resulted in the death of reproductive cancer cells (Shtam et al., 2013). RAD51 gene knockdown exhibited the same effect on HeLa cells and HT1080 cells. Exosome absorption by these two kinds of cancer cells seems comparable. According to the published results, exosomes loaded with siRNA through chemical loading were unsuccessful because the surplus of micelles (siRNA encapsulated in lipid micelles) could not be eliminated. Exosomes or an excess of micelles may transport the nucleic acid of interest to cells, but this is uncertain; consequently, the long-term use of this technique *in vivo* does not guarantee the safety of siRNA administration through exosomes. Exosomes were electroporated with siRNA targeting RAD51 to test this hypothesis. Electroporated exosomes were used as a test system to enhance HeLa cell transfection. Electroporation was utilized to insert heterologous siRNA into exosomes; however, the method may need to be modified for each exosome and cell type.

### Dendrimers as siRNA carriers

For the last several years, dendrimers have been actively researched as possible nucleic acid carriers (Boas and Heegaard, 2004; Cheng et al., 2008). It refers to macromolecules having highly symmetric, hyperbranched, spherical architectures, a broad range of molecular sizes, and variable surface charges. Due to its unique structural features, including chemical homogeneity, the possibility for enhanced production by repeating attachment of chemical groups, and a high surface density of functional groups, dendrimers are a promising polymer candidate for various biological applications. As medication and gene carriers, PAMAM (Polyamidoamine) and PPI (Poly-propylene imine) dendrimers, as well as other polycationic dendrimers, have been extensively investigated (Eichman et al., 2000; Gao et al., 2008). Dendrimers’ promise as a siRNA delivery vector, on the other hand, is yet largely untapped.

Cationic amphiphilic dendrimers (Yu et al., 2012; Liu et al., 2014a; Liu et al., 2016) have recently been developed that combine the multivalent cooperativity of dendrimer vectors with the self-assembling property of lipid vectors, thus capitalizing on the advantages of both lipid and dendrimer vectors for efficient delivery of therapeutic agents. When it comes to siRNA distribution, a multivalent self-assembling dendrimer is known as AD (amphiphilic dendrimers) performs very well for many cell types, including human primary and stem cells (Liu et al., 2014a). It's also worth noting that AD can transport siRNA to tumors in xenograft mice for effective gene silencing and powerful anticancer action *in vivo* (Liu et al., 2014a).

Dong et al. (2018), to further enhance AD-mediated siRNA delivery, aimed to provide AD the potential to target cancer cells specifically inside tumor lesions. By attaching a targeting moiety to the delivery system, active targeting may be achieved by interacting and binding to cell-surface receptors and/or molecules known as ligands (Bertrand et al., 2014). Thus, the therapeutic cargo may be delivered directly to the target cells, resulting in enhanced therapeutic effectiveness while sparing other cells from toxicity to achieve this goal. To build active targeting systems, several targeting agents, including antibodies and tiny molecule ligands, have been used (Davis et al., 2010; Wong et al., 2014). With its ability to target both tumors and healthy cells in one short section, the RGDK peptide is an excellent cancer-targeting peptide (Sugahara et al., 2009). On the one hand, RGD (arginine-glycine-aspartic acid) may interact with the overexpressed v3 integrin in tumor vasculature to target tumor endothelium; on the other, RGDK can bind to the neuropilin-1 (Nrp-1) receptor on tumor cells, facilitating cancer cell penetration and uptake (Teesalu et al., 2009; Desgrosellier and Cheresh, 2010; Marelli et al., 2013).

Using a poly (amidoamine) PAMAM dendrimer of generation 5 (G5) decorated with the same targeting peptide, researchers previously demonstrated the validity of this dual targeting method for siRNA delivery and improved performance in gene silencing (Liu et al., 2014b). PC-3 prostate cancer cells have integrin and neuropilin-1 receptor molecules on their surface, and RGDK was able to bind to and interact with these molecules. As a consequence, the siRNA was able to evade immune cells more effectively, resulting in a more robust anticancer impact in castration-resistant prostate cancer models both *in vitro* and *in vivo*, as compared to the non-targeted system. In addition, the targeted delivery method did not cause *in vitro* cytotoxicity, acute *in vivo* toxicity, or generated *in vivo* inflammation. Results from this study show that siRNA delivery using this targeting technique may result in gene silence with an anti-cancer effect.

Nanocarriers based on lipid-triblock PAMAM have recently been created for the co-delivery of small molecule drugs and siRNAs (Biswas et al., 2013). According to the findings, these hybrid nanocarriers were more efficient in loading doxorubicin and capturing siRNA in cells. For high-risk HPV-induced cervical cancer, PAMAM-nanodiamond hybrid nanocomplexes were used for the delivery of E7 or E6 oncoprotein-suppressing siRNA (Lim et al., 2017). According to the findings, the hybrid nanocomplexes demonstrated very minimal cell cytotoxicity and substantial suppressive effects. When PAMAM-hybrid was taken up by cells, the pH and mechanical stress of endosomes were lowered, allowing for the release of the siRNA.

### Nanoparticles

Traditional siRNA delivery mechanisms may be replaced with cyclodextrin. When combined with different payloads, the cyclodextrin may create inclusion complexes that have hydrophobic interiors and hydrophilic exteriors. Cyclodextrin’s tiny size, safety profile, and cationic nature make it an ideal delivery method for siRNA. However, adamantane, a hydrophobic molecule, is employed as an inclusion in the cyclodextrin cavity for higher transfection effectiveness (Bellocq et al., 2003). Targeted delivery of gene therapies against cancer cells was made possible by conjugating transferrin to the adamatane-cyclodextrin inclusion complex (Davis, 2009). For the first time, a targeted siRNA therapy was tested in humans using a cyclodextrin-polymer nanoparticle. As a targeted ligand, cyclodextrin, PEG, and human transferrin (Tf) were included in this nanoparticle system (CALAA-01). CALAA-01 effectively delivered siRNA targeting the RRM2 M2 subunit to human cancer cells overexpressing the transferrin receptor. CALAA-01’s safety profile in humans was examined in this phase I of clinical research (Zuckerman et al., 2014). For the most part, research on cyclodextrin-siRNA nanoparticles has been focused on *in vitro* effectiveness.

Hydrophobicity and transfection efficiency with siRNA has been improved by modifying chitosan with carboxymethyl groups Carboxymethyl chitosan, a pH-sensitive polymer, aided in the distribution of siRNA from a liposome formulation using pH sensitivity (Yao et al., 2015). To deliver siRNA/drug combinations into cancer models, hydrophobically modified glycol chitosan was used (Yoon et al., 2014) in the conventional method. Sequential administration of doxorubicin and BCl2 siRNA with 5-cholanic acid-modified glycol chitosan nanoparticles resulted in significantly improved anti-cancer activity over the long term. Other polymers and chitosan combine to generate hybrid nanosystems that boost transfection effectiveness for siRNA-based cancer treatment (Li et al., 2014; Xie et al., 2014; Zhong et al., 2015).

An excellent transporter of therapeutic nucleic acids *in vitro* and *in vivo* is magnetic iron oxide nanoparticles. Simple chemical approaches may be used to make these nanoparticles smaller (Reddy et al., 2012; Narsireddy et al., 2014). Nanoparticles’ surfaces may be coated with a variety of polymers that can assist in covalently or non-covalently attaching siRNA or DNA and prevent degradation. With the addition of polymers, such as chitosan or PEI (Polyethylenimine), magnetic iron oxide nanoparticles have a net positive surface charge, which aids in the electrostatic interaction with siRNA (Liu et al., 2011; Wang et al., 2016). Furthermore, these polymer coverings have many ligand-binding sites. Magnetic nanoparticles, such as iron oxide nanoparticles, can contain therapeutic siRNA, as well as the ability to display super paramagnetism (SPIO) when an external magnetic field is applied. An external magnetic field was used to transfect DNA using PEI-coated superparamagnetic nanoparticles *in vitro* and *in vivo*, as described by Scherer et al. The non-viral vectors often take longer to do focused transfections than viral vectors. It was later established that magnetofection may be used for the delivery of small interfering RNA (siRNA) into HeLa cells (Scherer et al., 2002). It has been shown that SPIONs may be used to minimize the accumulation and toxicity of siRNA in healthy tissues when they are delivered intravenously, directed by an external magnetic field (Arora et al., 2013).

IOMNPs may be used as a combination imaging and delivery technique for siRNA in breast cancers in mice, according to new research (Kumar et al., 2010). For NIR (Near-Infrared) imaging, IOMNP cores coated with dextran are triple-conjugated to peptide sequences (which selectively bind to tumor-specific antigen uMUC-1), the dye Cy5.5, and siRNA molecules (that binds the tumor-specific antiapoptotic gene BIRC5). Nano drug MN-EPPT-siBIRC5 was subsequently intravenously administered into subcutaneous mice models containing human breast cancers, and the *in vivo* quantitative MRI indicated that after 24 h of injection, the tumor T2 relaxation times were significantly decreased. NIR imaging indicated that MN-EPPT-siBIRC5 was preferentially taken up by the tumor, and a substantial reduction in tumor growth rate was seen as well. Clinical trials might be carried out using the IOMNP-based siRNA delivery method since various IOMNPs have previously been authorized.

IOMNPs’ theranostic function has also been shown in several other research, including those mentioned above. As an example, Lee et al. (2009)created a multifunctional manganese-doped IOMNPs (MnMEIO) coated with bovine serum albumin, which has a PEG decorated cell-specific targeting ligand (cyclic RGD peptide) and a fluorescent dye (Cy5) labeled siRNA (siGFP). In this work, breast cancer and lung cancer cells were employed as two different cell lines and treated with MnMEIO-siGFP-Cy5/PEG-RGD nanocomplexes, respectively. Increased 1/T2 signal strength in breast cancer cell lines with increasing quantities of the nanoparticles was seen in MR imaging of breast cancer cell lines. Lung-carcinoma cell lines did not show any difference, in contrast, indicating that these RGD-labelled nanocomplexes can only be taken up by cell lines expressing integrin. Fluorescence imaging of the Cy5 dye and GFP expression level were used to track the subcellular distribution and knockdown efficacy of MnMEIO-siGFP-Cy5/PEG-RGD nanocomplexes. In all, the findings showed that an imaging-guided siRNA distribution technique might provide prospective advantages, including therapeutic treatment and visualization for *in vivo* and future clinical investigations simultaneously.

Genes that cause resistance to chemotherapeutic medicines like cisplatin, paclitaxel, and doxorubicin when overexpressed are another category of possible targets (Bao et al., 2017). The MDR gene product P-glycoprotein (P-gp), encoded by ABCB1, was the focus of Yang et al. (Yang et al., 2015). Cancer cells overexpressing the cell surface protein CD44 were targeted with HA nanoparticles containing siRNA against ABCB1. P-gp levels were reduced for up to 120 h after HA-NP administration in OVCAR8TR paclitaxel resistance cells, indicating that the introduction of HA might be employed as an effective therapy to restore cells’ ability to respond to paclitaxel treatment. Treatment was given intravenously to mice with human MDR OVCAR8TR tumor xenograft models, and the animals were observed for 35 days. In HA-PEI/HA-PEG nanoparticle-treated groups, the expression of the target gene is reduced and tumor development is inhibited (Yang et al., 2015).

A transcription factor known as TWIST, which is linked to inducing chemotherapy resistance and cancer cell stemness, is also linked to ovarian cancer medication resistance (Vesuna et al., 2009). As a result, cancer cells are more susceptible to chemotherapeutics when TWIST is down-regulated, according to research (Finlay et al., 2015a). The siRNA targeting TWIST (siTWIST) was delivered to cisplatin-resistant ovarian cancer cells using PAMAM dendrimers and mesoporous silica nanoparticles (A2780R) (Roberts et al., 2017). They predicted that knocking down TWIST would cause inducing chemotherapeutic resistance. To confirm that the dendrimer-siTWIST combination was absorbed by A2780R cells, researchers employed fluorescence microscopy. After 1 week of treatment, Western blots showed that the siRNA therapy effectively reduced the TWIST protein product relative to the nontargeting siRNA and untreated controls, indicating that the siRNA treatment was effective (Finlay et al., 2015b). Si419H with cisplatin reduced tumor weight in mice treated intraperitoneally with OVCAR8 cells sixfold, as evidenced by *in vivo* tests. TWIST was shown to be a viable target for siRNA treatments and nanoparticle delivery in this study (Roberts et al., 2017).

Using a gold nanoparticle-based aptamer-siRNA chimera delivery system, Chen et al. set out to inhibit the expression of NOTCH3, which encodes a marker for ovarian cancer recurrence and treatment resistance (Chen et al., 2017). An overexpressed form of vascular endothelial growth factor (VEGF) was discovered to behave as a receptor for the gold nanoparticles, which were then combined with ferric (II, III) oxide (Fe_2_O_3_), PEI, and an aptamer-siRNA to target ovarian cancer cells (Cho et al., 2012). Due to its positive (+20 mV) electrical charge, the aptamer could engage with the negatively charged cell membrane and enable clathrin-mediated endocytosis more quickly than if it had been neutrally charged (Kaksonen and Roux, 2018). When the aptamer was used in combination with the nanoparticle-chimera delivery method, it was shown to be more efficient than lipofectamine or siRNA alone in cisplatin-resistant SKOV-3/DDP cells in knocking down NOTCH3 expression. In addition, SKOV-3/DDP cells’ viability was reduced by a factor of two when compared to the untreated control. It seems that this aptamer-siRNA chimera delivery method is an effective method for reducing ovarian cancer medication resistance via the use of nanoparticles (Chen et al., 2017).

Nanoparticles of chitosan and siRNA were utilized to inhibit P-gp expression in the blood-brain barrier by Malmo et al. (2013). As a result of this method, the flow of medications from the blood to the brain would be improved. To test this theory, the researchers employed a type of rat brain endothelial cell line, RBE4, often used as a BBB model to transfect siRNA against P-gp. However, the effectiveness of the PEC nanoparticles to silence P-gp was dependent on the N/P ratio employed to produce the chitosan/siRNA nanoparticles. Increasing the N/P ratio lowered the quantity of internalized siRNA given by PEC nanoparticles, but high N/P ratios were required to achieve the greatest knockdown of P-gp expression. Success in transfecting RBE4 cells with siRNA showed that P-gp expression had been reduced *in vitro* and that efflux capability had decreased. As a model drug, doxorubicin was shown to be more effectively administered to the cells, resulting in an increased level of bioactivity. The authors’ findings imply that transfection of a siRNA targeted to quiet P-gp into blood-brain barrier endothelial cells might be utilized as a general technique to increase medication delivery to the brain, which is still a bottleneck for effective therapy for many kinds of disorders (Malmo et al., 2013). The ability of siRNA/chitosan PEC nanoparticles to suppress the expression of the P-gp and boost the efficacy of active delivery in the brain will only be completely shown *in vivo* investigations. In addition to doxorubicin, several other relevant medications will need to be considered for delivery to the brain as part of the method’s validation.

Using chitosan/siRNA PEC nanoparticles, Fernandes et al. (2012) studied the *in vitro* transfection efficacy of a siRNA directed against the Sjogren Syndrome antigen (SSB, GenBank accession number NM009278) on cell lines from cervical cancer, ovarian cancer, and osteosarcoma. In contrast to the third cell line, MG-63, two of the cell lines tested, HeLa and OV-3, expressed folate receptors on the cell membrane. A nanoparticle targeting strategy was tested by making chitosan nanoparticles containing folic acid residues to see how effective it was. It was hypothesized that including a targeting ligand in the siRNA delivery system would enhance the siRNA’s transfection efficiency in the cells that would be receiving it. Folate chitosan/siRNA PEC nanoparticles improved transfection efficiency in two folate receptor-positive cell lines, HeLa and OV-3. Targeting folate receptor-expressing cancer cells using nanoparticles coated with folate groups on their surface was the goal of this research. Contrary to what was expected, the presence of the targeting ligand on the PEC nanoparticles did not influence MG-63 cells’ ability to transfect siRNA (Fernandes et al., 2012). HeLa cells were utilized to test the cytotoxicity of various molecular weights of chitosan employed in the manufacture of chitosan/siRNA PEC nanoparticles. In comparison to chitosans of 10, 25, and 50 kDa, the chitosans of 2, 5, and 10 kDa are more cytotoxic (IC_50_ = 0.21 mg/ml). Chitosans 10 and 25 kDa had the lowest cytotoxicity, with an IC_50_ of 2.2 mg/ml. The cell survival was unaffected by the chitosan/siRNA ratio in the PEC nanoparticles up to a ratio of 100 when using chitosan with a molecular weight greater than 10 kDa. Furthermore, the decreased cytotoxicity of the targeted siRNA delivery method to folate receptor-positive cells was associated with the better transfection efficiency obtained with the folate-chitosan nanoparticles. If the folate receptor overexpression is seen in multiple forms of cancer and inflammatory disease-causing cells such as those found in arthritis, this should spur more research into cell-specific delivery of siRNA.

It has been shown that nanospheres coated with chitosan may deliver and preserve siRNA directed against an oncogene implicated in radio-induced thyroid cancer tumorogenesis, such as RET/PTC1 (De Martimprey et al., 2008). A significant level of suppression of the RET/PTC1 junction oncogene expression was seen when the shRNA was transfected into cells using lipofectamine and even reversed the phenotypic of NIH/3T3 cells containing the RET/PTC1 oncogene. For *in vitro* investigations, lipofectamine was chosen as a transfection agent due to the lack of suppression of the target gene when PIBCA nanospheres were transfected with siRNA-loaded chitosan. *In vitro*, the nanosphere-carrying siRNA was completely ineffective in suppressing the expression of the target gene, but intratumoral delivery of the siRNA with these nanospheres resulted in a considerable suppression of tumor development (De Martimprey et al., 2008). It was shown that the chitosan-coated PIBCA nanospheres were effective in protecting siRNA from nuclease degradation in tumoral tissue over 48 h, pointing in the direction of the antitumoral activity hypothesis. Nanospheres were also able to transport the siRNA to cancer cells, based on their ability to disrupt cancer cell interferon activity.

As part of the first investigation, a siRNA was delivered that was meant to halt the production of the green fluorescent protein gene. For 5 days, patients received chitosan/siRNA PEC nanoparticles intranasally every day (Howard et al., 2006). A decrease in green fluorescent protein expression in lung bronchiole epithelial cells was seen even though the medication was well tolerated and no overall harm was noted. When compared to the expression levels in untreated animals, the inhibition percentage was 43%. Chitosan/Cy3-labeled siRNA PEC nanoparticles were aerosolized using an intratracheal catheter and inhibited the production of green fluorescent protein by 68 percent in comparison to control tests by Gianfelici et al. (2012). Using PEC nanoparticles, siRNA can transport to the lung in an active state. They have a positive outlook on the findings, citing applicable lung disease models as an example.

### Specific targeted delivery

siRNA had been combined with the chimera and aptamer for the cell specific delivery of siRNA. A key benefit of this technology is that it uses just RNA (in the form of an RNA aptamer coupled to a siRNA), rather than proteins or other regularly used reagents, which have several adverse effects. Aptamer siRNA chimeras do provide various benefits for *in vivo* use. The immunogenicity of aptamers and siRNAs is minimal. Large amounts may be produced cheaply, and chemical changes can be made to increase their resistance to degradation and enhance their pharmacokinetics in the body. Aptamers are easier to distribute *in vivo* because of their reduced size compared to antibodies, which allows for greater tissue penetration. The PSMA-specific aptamer–siRNA chimeric RNAs were created to target siRNAs to cells that express PSMA on the cell surface. Binding between PSMA and A10 (aptamer) occurs via this component. PLK1 (A10-Plk1) and BCL2 (A10-BCL2) are the two survival genes targeted by the siRNA component (A10-Bcl2). They used RNAi to target anti-apoptotic genes in cancer cells that expressed the cell-surface PSMA receptor (McNamara et al., 2006). Targeted cell growth and apoptosis were reduced after depletion of the targeted gene products. The aptamer part of the chimeras interacted with PSMA on the cell surface to help them target cells with the cellular RNAs.

A mutant chimeric RNA with two-point mutations in the aptamer region responsible for binding to PSMA lost its binding activity. It was shown that chimeras targeted LNCaP cells that had been depleted of the PSMA protein by 5-adihydrotestosterone treatment, but not PC-3 cells or LNCaP cells that had not been depleted of PSMA. Additionally, PSMA-specific antibodies competed with the chimeras for binding to the LNCaP cell surface. Using monoclonal antibodies and RNAi-mediated gene silencing, Song and others present a novel technique for delivering siRNA to cells (Dassie et al., 2009). With the F105–protamine method, siRNA may be efficiently transported across the cell membrane and targeted to certain cell types. The avoidance of silencing in cells not implicated in the particular illness is one conceivable advantage of a tissue- and cell-type specific administration. This should increase the number of genes that can be targeted using a siRNA therapeutic strategy to non-target cells. In addition, administration to certain structures (such as tumor cells) may limit the activation of the interferon pathway, which has been documented for siRNA delivery to plasmacytoid dendritic cells when confined to those structures alone. Jiang et al. used quantum dots (QDots) to identify HA derivatives and studied the impact of HA (Hyaluronic acid) alteration on receptor-mediated endocytosis (Song et al., 2005). Endocytosis mediated by the HA receptor seemed to take up HAQDot conjugates with a degree of modification less than around 25 mol percent more effectively than QDots alone in B16F1 cells. Target-specific intracellular delivery of siRNA was established based on bioimaging studies using polyethyleneimine, PEI-HA compound with 24.2 mol percent PEI concentration. In B16F1 cells expressing HA receptors, the siRNA/PEI-HA combination suppressed more genes than the siRNA/PEI complex alone. According to the results, the PGL3-Luc gene was silenced between 50% and 85%, depending on the serum content of the siRNA/PEI/HA combination. siRNA/PEI-HA combination accumulated mostly in organs having HA receptors, such as liver, kidney, and tumor, according to *in vivo* biodistribution tests. Furthermore, intratumoral injection of anti-VEGF siRNA/PEI/HA complex resulted in an efficient reduction of tumor development in C57BL/6 mice through the HA receptor-mediated endocytosis to tumor cells (Jiang et al., 2009). Similarly, Cao et al. (2019) studied the effect of pH responsive micelles based on siRNA for the targeted delivery in hepatocellular carcinoma.

## Conclusion and future directions

Despite preclinical and clinical research showing improvement, siRNAs as cancer treatments face hurdles. Unsolved concerns include siRNA’s stability in blood and tumor microenvironments, selective absorption into tumor cells, immunological adverse effects, carrier toxicity, and commercialization hurdles. Initially, chemical changes to siRNAs were thought to be required for *in vivo* application. Several siRNA alterations impair RNAi activity and therapeutic index. To address siRNA’s lack of tumor-site accessibility, delivery techniques that increase tumor cell uptake are needed. New delivery materials were expected to render chemically modified siRNAs uneconomical. If cost limits can be addressed, combining delivery modalities with chemically modified siRNAs may improve siRNA stability in blood and tumor microenvironments.

In most siRNA-based anti-cancer studies, just one gene has been targeted. siRNA monotherapy has modest anticancer effects despite suppressing the target protein in malignant tissues. Due to cancer’s polygenic pathogenesis and existing therapy, silencing a single protein is unlikely to eliminate this complex and persistent organism. The ALN-VSP02 strategy, which uses dual siRNAs to target two tumor-essential proteins, may be more practicable for clinical studies. Anticancer siRNAs may be more effective when used with chemotherapy. Anticancer siRNA may be used as a chemosensitizer or a radiation-sensitizer to make resistant cancers more sensitive to treatment. Most siRNA nanoparticle delivery techniques rely on the EPR effect to enhance tumor accumulation. EPR effects may boost siRNA nanoparticle distribution to organs including the liver, lung, spleen, and kidney, whereas unbound siRNAs may be delivered more effectively to tumor tissues. Numerous research has exclusively concentrated on silencing siRNA target genes in tumor tissues, disregarding other tissues. Before using siRNA to quiet a target gene in other places of the body, clinical studies must detect any negative effects. siRNAs introduced into the tumor microenvironment don’t always reach tumor cells. A siRNA ligand might be added to delivery systems to boost cancer cell absorption.

According to past studies, ligonucleotides may improve tumor cell absorption rather than siRNA delivery. This is exciting, but it raises questions about whether ligand change affects animals and people similarly. Extrapolating ligand-modified delivery system results in human trials requires care. Anticancer siRNAs must be able to infiltrate tumor tissues for delivery strategies to be effective. After accessing tumor tissue, a new medication delivery system is contracted. Enzymatic breakdown of 100 nm nanoparticles into smaller nanoparticles in the tumor environment improves penetration. Future therapies may combine chemically modified siRNAs with tumor microenvironment-sensitive delivery techniques. RNA interference is less damaging than standard chemotherapeutics and is used to treat cancer. siRNAs may heal genetic faults, skin ailments, cardiovascular difficulties, infectious illnesses, and malignancies. If protein-based medications or comparable small molecules can’t be employed, siRNA may help treat cancer. siRNA in cancer therapy is safe, efficient, flexible, and precise. Many methods for delivering siRNA have been developed. The design, size, and content of effective delivery systems vary greatly, however, there are criteria for focused distribution. Nanoparticle distribution networks should include particles between 20 and 200 nm to avoid renal filtration and impair phagocytosis clearance.

Exogenous or endogenous trigger-binding affinity helps cancer cells absorb siRNA. Despite research showing siRNA’s promise in cancer treatment, this revolutionary drug delivery technique hasn't reached its full potential. Future studies should concentrate on the safety and effectiveness of nanoparticle delivery techniques with unwanted cytotoxicity and immune activation, such as composites, cationic lipids, dendrimers, and inorganic NPs. SiRNA-based cancer treatment relies on easy approaches for defining rules and evaluating biocompatibility, biodegradability, and environmental safety in clinical trials. SiRNA-based therapies need an effective delivery mechanism. Due to advances in siRNA delivery techniques, the siRNA drug sector, especially the chemotherapeutic agent industry, is in a strong position after this breakthrough.
